# Report of Committee on Intelligence and Education

**Published:** 1894-11

**Authors:** Olof Schwarzkopf

**Affiliations:** Phila., Pa.


### REPORT OF COMMITTEE ON INTELLIGENCE AND EDUCATION.

31ST ANNUAL CONVENTION UNITED STATES VETERINARY MEDICAL ASSOCIATION.

PHILA., PA., Sept., 1894.

With specific reference to faculty, buildings, apparatus, clinical advantages and post-mortem cases, what constitutes a Veterinary College?

A Veterinary College should have full Professors at the head of the following departments:

1. Anatomy, Comparative and Special; 2. Pathology, Histology and Bacteriology; 3. External Conformation of the Horse and the Principles of Breeding; 4. Veterinary and Operative Surgery; 5. Veterinary Medicine; 6. Materia Medica, Therapeutics and Pharmacology; 7. Obstetrics and Cattle Practice; 8. Physiology and Opnthalmology; 9. Zoology, Helminthology and Botany; 10. Chemistry and Toxicology; 11. Meat and Dairy Inspection and Sanitary Science; 12. Shoeing; 13. Canine Practice; 14. Physics and Latin.

Each department Professor should be a specialist. He should also have ability and experience in teaching. It is a well known fact that some men may possess fine technical ability and skill, but be wanting in ability to teach, to impart knowledge to others. Good instructors and technical experts may be hard to find, nevertheless they are the best men for any kind of a college faculty. Such men may be obtained by competitive examinations, providing the examining boards understand their duty.

It is also requisite that each department be supplied with a sufficient number of competent assistants.

Furthermore, no department, excepting chemistry, physics, botany, zoology and possibly bacteriology, should be delegated to a man who has only graduated in human medicine. Veterinary pathology and histology, veterinary materia medica and therapeutics, and veterinary physiology should no longer be taught by human practitioners, but by Veterinarians. Many of this Association, who have graduated at institutions where some of the faculty were practical M.Ds. and theoretical Veterinarians, will bear me out in saying that such men are not qualified to become expert professors in Veterinary Medical Colleges.

Each member of the faculty should not only be a practical expert and a good instructor, but also an investigator. His work should be accurately recorded, and the accumulated facts or results given to the profession, from time to time, for the improvement of veterinary science and the advancement of veterinary education.

The course of study should not embrace less than three years of nine months each. In truth, with the low entrance examination required by the American Veterinary Colleges, the course ought to cover four or five years of nine months each. The hot-bed system, as practiced at all the two year colleges, forces nature. Unless the individual natural element is well cultivated (educated) before entering one of these medical hot-beds, the result is nearly always a hybrid ; a cross between a quack and a scientific veterinarian.

Moreover, the course of study should be graded. Text books should, as a rule, be used as reference books, except in such cases where it is possible to use the text book in class, and supplement the same by lectures; but graded courses of didactic lectures, with frequent written and oral reviews, are, as a rule, the best.

In regard to buildings, a Veterinary College can not have too many nor can they be too large. A glance at the catalogues of nearly all the American Veterinary Colleges, will show immense and numerous "paper" buildings ; but how insignificant the real buildings are when compared with those from the hand of the engraver and the pen of the catalogue writer. If all the buildings and space at the command of every Veterinary College in the United States were united, or assembled in one place, the whole would scarcely exceed, if equal, the capacity of the Berlin or Alfort school. Veterinary Colleges cannot be successfully conducted with small and few buildings and limited space. In fact, every department should have its special building, and such departments as surgery, internal medicine, canine and cattle practice, should be abundantly supplied with hospital wards, rooms, stalls and exercise grounds.

The apparatus necessary for a complete Veterinary College forms no small item. The chemical, the pathological, the histological, the bacteriological, the zoological, the botanical and the pharmaceutical apparatus in most American Veterinary Colleges is not what it should be. In fact, short sessions and two year courses prohibit anything like laboratory practice, and limited means and want of room prevent the appearance of much apparatus. Consequently very few medical men in America learn anything but theoretical chemistry, bacteriology, histology and pathology, unless they get them before entering a medical college, or in post graduate courses.

In reference to clinical cases, it is beyond question that each department cannot have too many. Each department should have daily at least one capital operation, case or demonstration and many minor cases. It is not enough to have one general clinic, each department should have its special clinic and make the clinic practical daily object lessons. Every professor should make his department as practical and real as the subject of equine anatomy is usually made. It is true that every department may not have from two to four thousand cases annually, or the pathological department have five hundred to one thousand post-mortem cases annually, but such numbers have been reached in Continental European Veterinary Colleges, and they can be in America, when we have colleges that are fully manned, properly equipped and backed by the States.

Someone may ask, what means this discussion on what a Veterinary College should be? It means that many, yea the majority, of the American Veterinary Colleges are in many respects defective; that there are reasons for this poorness of quality and littleness in quantity, and that there is a remedy which may be suggested, if not carried into effect.

During the last five years numerous little, private Veterinary Colleges have been thrust upon the American public. Such institutions as the Des Moines Veterinary College, the Detroit Veterinary College, the Ohio Veterinary College, the Kansas City Veterinary College, the New York College of Veterinary Surgeons, the U. S. Veterinary College, the National Veterinary College and the Washington Veterinary College are not, and never were, pressing necessities. In fact, most of them are children of misguided parents, and ought never to have been born. The Chicago Veterinary College has graduated too many illiterate and poorly qualified men, and some of her graduates never attended any college, but the Chicago Veterinary College, one session from four to six months in duration. This College may be doing better now, but it still needs more good men in its faculty, more buildings, space, apparatus, longer sessions and a course not less than three years in dnration.

I also question the demand for the new McKillup Veterinary College. In fact, unless its endowment is greater than has yet been made manifest, I sincerely question the starting of another private school to depend upon patronage for support.

The American Veterinary College, of all the private schools in America, has done the best work, but it is yet sadly lacking in buildings, space, apparatus and departments.

The Ontario College, at Toronto, is grinding out too many two year men. It needs reorganization on a broader and more extended basis ; it needs more apparatus, more departments and some new blood in its faculty.

The Montreal School has started out along the right line, and bids fair to become an institution worthy of patronage. However, it is lacking in many things when compared with a complete veterinary college.

The State veterinary institutions are not all fine models of complete veterinary colleges.

The Veterinary Department of the University of Ohio is not sufficiently equipped with veterinarians, buildings, apparatus, and clinical cases to justify the granting of veterinary degrees. The Veterinary Department of the Iowa Academy and Medical College needs more buildings, more veterinarians in its faculty, and more clinical cases. The Veterinary Department at Cornell has been sadly deficient in buildings, in the number of veterinarians, in its faculty and in apparatus. Harvard also needs more veterinarians, more full-fledged departments, more buildings and apparatus.

The Veterinary Department of the University of Pennsylvania is the only State veterinary institution that stands on what we may term a fair footing. Its prospects are better than any other veterinary school in America. It is backed by the State, as all veterinary colleges in the United States should be, yet it needs many improvements to put it on an equal with European schools. It should have more veterinarians, more apparatus and more buildings.

It is true that the private schools of this country have done the cause of veterinary science and education considerable good; but that does not prove that they have not indirectly done some harm.

With due deference to private schools and their supporters, many of whom fought bravely and manfully for the cause during the pioneer days of veterinary education in America, I firmly believe it would have been, or would be, better had there never been a solitary private veterinary college inthe United States. No one has opened his philanthropical heart and liberally endowed any of the private veterinary colleges, as has been done with human medical colleges and other institutions of learning. The fact is, all the private schools have been battling for existence, and many of them have done shady things for patronage. During this time the existence of these private institutions has prevented States from establishing veterinary colleges in connection with their respective universities. Note the fact that there are no private colleges of any note in Iowa or Pennsylvania, where State schools are flourishing. Also compare the Continental government veterinary colleges with the private veterinary colleges of England and Scotland. As far as I can determine from the history of veterinary institutions, no private veterinary college has ever succeeded in a marked degree. Again, look at the history of human medical colleges in this country. The State schools were the first to lengthen the course from two to three or four years, and the session from five months to nine. And, as a rule, the State medical schools are doing the best work.

Furthermore, we must remember that all public educational institutions are controlled and regulated by the States in which they are located. Confidently, I believe it is the duty of every member of the United States Veterinary Medical Association to encourage his respective State to build and equip a State veterinary college, and grant no charters to private schools unless they equal the State colleges in faculty, buildings, equipment, and course of study.

Finally, we must remember that the respective States may or can charter schools, and the United States Government will not prohibit, at least the general Government has never interfered with, State schools. Hence, if we wish uniformity, thoroughness and completeness, we must obtain them by State enactments.

C. A. CARY, Chairman.

DR. C. A. CARY, Chairman, Committee on Intelligence and Education:

As a member of the Committee on Intelligence and Education, I have the honor to submit the following views on the topics I was requested to discuss:

Topic 1st—"Shall the Veterinary Colleges Require an Entrance Examination in English Grammar, United States History, Geography, Writing, Arithmetic, Orthography and Civil Government?"

At the present day few would answer this question in the negative. We say decidedly yes. Possibly Civil Government might be omitted, but as the student can hardly possess a fair knowledge of the other subjects without knowing something of the last, it may be included. We answer yes for two reasons. First, unless the student possess a knowledge of the subjects named, he is not prepared to carry on the work as taught in a first-class veterinary college. All through the course he will be hampered by his want of primary education, and not only will the work be harder for him, but, may he be ever so studiously inclined, he cannot accomplish what might be easily done by one possessing a good knowledge of the branches named. For example, with only a little knowledge of Arithmetic, how can the chemical problems which arise be solved? Again, without a fair knowledge of Composition and Grammar, how can the student intelligently express himself especially when his thoughts must be put on paper? Many other examples might be cited. My experience has taught me that veterinary students are more deficient in language than in any other branch. Second—Not only does the student need a knowledge of these subjects so that he may be capable of carrying on the work to the best advantage to himself, but so that his educational qualifications after graduation will place him on a level with men in other professions. In other words, if educated, the veterinarian makes a better appearance and secures a much better standing in the community in which he lives. We all know that it was the extreme ignorance of the now fast becoming extinct "horse doctor" that brought disrespect on the veterinary profession, and that when educated men entered the profession its standing advanced.

We believe that a sufficient number of veterinary students who can pass good examinations in the branches named can now be secured to fill the demand for veterinarians, and that the old plea of "scarcity of veterinarians justifies no entrance examination" (made by a short-term college last year at the Chicago meeting) is no longer justifiable. We also believe that in the near future it will be found advisable to either raise the qualifications for entrance, or make the course sufficiently long to give time for some subjects which would greatly assist the student in acquiring a knowledge of the purely veterinary studies.

This brings us to a discussion of:

Topic 2d—"Should the Veterinary Colleges Require an Attendance of Three Years of Not Less than Twenty-Six Weeks Each?"

To this question I also answer yes. Three sessions of twenty-six weeks each is not a particle too much time in which to acquire a good knowledge of veterinary medicine and surgery. We would say that the time could be lengthened to better advantage than shortened. A shorter period of time necessitates great neglect of most of the correlative subjects pertaining to veterinary science, and omission of some subjects of considerable importance which can be included when the curriculum extends over a longer time. A perusal of the catalogues of the two and three year schools will prove the correctness of these statements. Of course, it is plain that if we could secure students with the B. Sc. degree, a shorter course would be sufficient, but a majority of students know nothing of medicine or its correlative subjects, being only expected to pass examinations of the subjects mentioned in Topic 1st.

While the adoption of a course of this length by all the Colleges on the continent would, no doubt, lessen the number of students slightly for a time, I believe such a step would greatly advance veterinary education in this country. It had been truly said that the profession has more to fear from the empiric with his diploma, than from the ignorant "horse doctor" without anything which tends to place him on a footing with qualified men; and what can the graduate of many short course colleges be called but an empiric?

We would add that, for the good of the student while at college, and after graduating, and for the proper advancement of the veterinary profession, the college course should not be shorter than three sessions of twenty-six weeks each.

Topic 3rd:—

"Should the State have jurisdiction over the course of study in State and private veterinary colleges within her borders, or has the State a right to control the work done by a corporate veterinary college within her borders? If so, should it be done at present on lines prescribed by the U. S. V. M A.?"

We believe the State should have jurisdiction over the course of study in all veterinary colleges within her borders. This would lead, or should lead, to an improvement in the curriculum of many colleges as now organized. We believe this would be for the "public good," and on this ground the State would have a perfect right to control work done by a corporate school. Such control would prevent, if properly carried out, the organizing of new colleges, which are intended only to secure fees and advertise their incorporators. It would prevent the graduation of incompetent men, and would thus prevent the flooding of the country with men whom the public would unhesitatingly employ, to the detriment of themselves and the qualified men of the State.

As to what jurisdiction the State should exercise I believe would depend largely on existing conditions. At present jurisdiction over the length of the course of study would be all that should be undertaken. While State control of all veterinary colleges within the borders of the State would not give uniformity in all colleges in the country, it would, no doubt, be a long step in advance of what we now have, and might prove a stepping stone to uniform veterinary education in this country.

W. B. Niles.

COMMITTEE ON INTELLIGENCE AND EDUCATION.

1. What are the objects and limits of veterinary instruction in agricultural colleges, and what credit should a veterinary college allow the agricultural graduate?

Veterinary science is essentially a medical science. While this is true as to its nature and purpose in its practical application and economical results, it is also related to agricultural science. This is one of the reasons why veterinary science, in its history, has found itself so often in close relationship with agriculture, in fact, oftener and closer so than it ever was approached by the other medical science.

If this is true, as it is, there ought to be a mutual friendship and regard between the veterinary and agricultural colleges. Each can learn from the other. Both of these classes of colleges have collateral branches of study: Botany, Zoology, Chemistry, the elements of Physiology and Anatomy, Stock Breeding and Feeding, the care of injured and sick animals, Hygiene, are justly subjects of instruction at agricultural schools. An attempt at a smattering of Pathology and Pharmacy means a stepping over the natural limits of agricultural science, and a trespassing upon a field of study that belongs altogether to a different science.

I know that this is not always realized by Boards of regents, trustees and other laymen, who generally formulate the course of study at our agricultural schools. Time, however, will prove the fruitlessness of such attempts, and these wrongs will right themselves on their natural basis.

In the face of the fact that the first course of study at veterinary and agricultural colleges contains so many collateral branches, all three year colleges can well afford to credit graduates of agricultural colleges with the first year. This, I think, is just. But it is unjust to ask for any more. The second and third year instruction in agricultural colleges branches off into a different field from ours, and the purely veterinary studies, which might have been taught there, could only have been so rudimentary and superficially that they cannot be considered equivalent to a medical instruction in veterinary schools. A permission, therefore, for advanced work should only be granted after an examination by the veterinary faculty, if at all.

On the whole, the student of an agricultural school will be found a welcome acquisition for any veterinary college. He is a rugged young fellow, sound in body and mind. Besides, he has got a touching up in English education, has acquired a habit of study, and above all he instinctively finds himself at home among all the different types of domesticated animals, which constitute our field of labor and usefulness as veterinarians.

2. What should the veterinary college allow the graduate in human medicine?

Human medicine is a special medical science, and veterinary medicine is a comparative medical science. The medical student becomes a specialist already in his study of anatomy. The more he advances, the more he seeks for specialties, and he ends his studies with the detailed study of the diseases of the eye, ear, nose, throat, diseases of women and children, etc.

Such specialties are out of the reach of the veterinary student. He has to devote his study to the anatomical, physiological and pathological peculiarities of six or more different types of domestic animals. This is all he can do at college, and any specialty he wishes to pursue he must develop in his later practical life.

There are many poor medical colleges in this country; some of them worse than our bad veterinary schools. No student or graduate of such medical school should be recognized or admitted to any of our reputable veterinary colleges.

From my acquaintance with the work of several of our high grade medical colleges, it is fair to conclude that any graduate of a four or three term medical school will need no longer than one session to acquaint himself with the peculiarities of the veterinary science. But by no means will he have an easy task. With fair safety the maxim can be established that the medical student at a veterinary college and the veterinary student at a medical college should be required to attend one additional year at the opposite colleges.

OLOF SCHWARZKOPF.

