# Error

**Published:** 1895-02

**Authors:** 


ERROR.—On page 385 in the November number of THE JOURNAL, referring to the veterinary department of the Iowa College, the words academy and medical should read agricultural and mechanical.

